# Surfactant-facilitated crystallisation of water-soluble foldamers†
†Electronic supplementary information (ESI) available: Detailed experimental procedures, additional figures and tables describing structures as well as crystallography details. CCDC 1050867–1050870. For ESI and crystallographic data in CIF or other electronic format see DOI: 10.1039/c6sc00090h


**DOI:** 10.1039/c6sc00090h

**Published:** 2016-02-10

**Authors:** G. W. Collie, K. Pulka-Ziach, G. Guichard

**Affiliations:** a Univ. Bordeaux , CBMN , UMR 5248 , Institut Européen de Chimie et Biologie , 2 rue Robert Escarpit , 33607 Pessac , France . Email: g.guichard@iecb.u-bordeaux.fr; b CNRS , CBMN , UMR 5248 , F-33600 , Pessac , France

## Abstract

X-ray crystallography has played a major role in the advancement of foldamer research, however, obtaining well-formed single crystals of suitable quality for structure determination by X-ray diffraction methods is often rather challenging. Towards this end, we report here the ability of common surfactants to promote the crystallisation of a series of water-soluble oligourea foldamers which had previously proven highly resistant to crystallisation. Four high-resolution crystal structures are reported, suggesting certain surfactants could be potentially useful tools for the crystallisation of intractable water-soluble foldamers (or peptides).

## Abstract 2

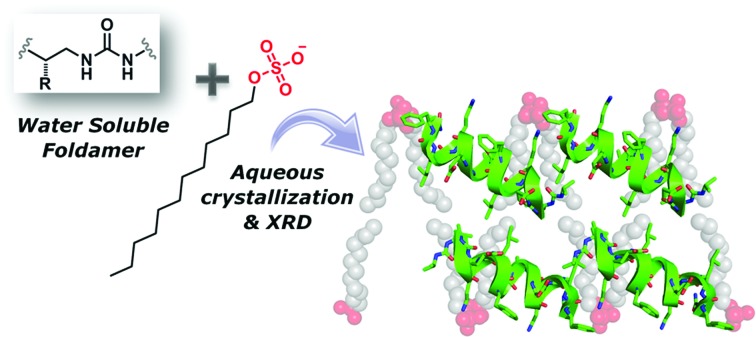
Common surfactants promote the crystallisation of a series of water-soluble oligourea foldamers which had previously proven resistant to crystallisation efforts.

## Introduction

There is considerable interest in the creation of artificial folded molecules able to mimic certain desirable qualities of natural biomolecules. Such molecules – termed foldamers^1^,^2^ – have been developed within the contexts of a broad range of applications, including biopolymer surface recognition,^3^–^8^ host–guest chemistry,^9^–^15^ catalysis,^11^,^16^–^19^ aqueous self-assembly,^19^–^25^ and nano-technology.^26^–^28^ X-ray crystallography continues to contribute considerably to the growth and progress of the foldamer field, permitting the structures and functions of novel foldamer architectures to be understood at the atomic level. The growth of single, well-ordered crystals suitable for structure determination by X-ray diffraction methods is consequently an important procedure in the foldamer field, however, this process can often be challenging, particularly with respect to the crystallisation of water-soluble foldamers using aqueous biocrystallographic methods (such as vapour diffusion). One factor which almost certainly contributes to the difficulty in obtaining well-ordered aqueous crystals is the typically high solvent contents of such crystals (up to 70%), which hinders the formation of strong intermolecular contacts within a crystal lattice. As one potential method to overcome this obstacle to aqueous crystal growth, we report here the use of cationic and anion surfactants as a means to crystallise a series of fully water-soluble foldamers which had previously proven to be highly resistant to crystallisation efforts. A series of short water-soluble aliphatic oligoureas bearing proteinogenic side-chains were crystallised in the presence of cetrimonium bromide¶
¶Also known as cetyltrimethylammonium bromide and hexadecyltrimethylammonium bromide. (CTAB) or sodium dodecylsulfate (SDS), permitting high resolution X-ray structures to be determined with resolutions ranging from 1.19 Å to 1.84 Å. Crystals of the oligoureas in the absence of surfactant could not be obtained – indeed, analysis of the crystal structures reveals the surfactant molecules to play a crucial role in crystal packing, forming key intermolecular packing contacts and thereby acting as ‘molecular glue’ in the crystal lattice. Currently, by far the most commonly reported use of surfactants in biocrystallography is as a means to aid the solubilisation of membrane-associated proteins^29^–^31^ (which naturally have limited solubility in water), with very few reports of alternative uses of such molecules.^32^,^33^ Although several innovative techniques for obtaining well-ordered single crystals of short oligomers including nucleic acids, peptides and foldamers have been reported – such as racemic^33^–^40^ and quasi-racemic^41^,^42^ crystallographic methods – to our knowledge, the use of surfactants as a means to facilitate the crystallisation of water-soluble yet otherwise difficult-to-crystallise foldamers or peptides has not been reported. We believe that the findings reported herein may be of interest to those engaged in recalcitrant aqueous foldamer (or peptide) crystallogenesis studies, as a possible means to generate well-formed crystals – and consequently atomic-scale details – of synthetic biomimetic molecules.

## Results and discussion

**Fig. 1:**
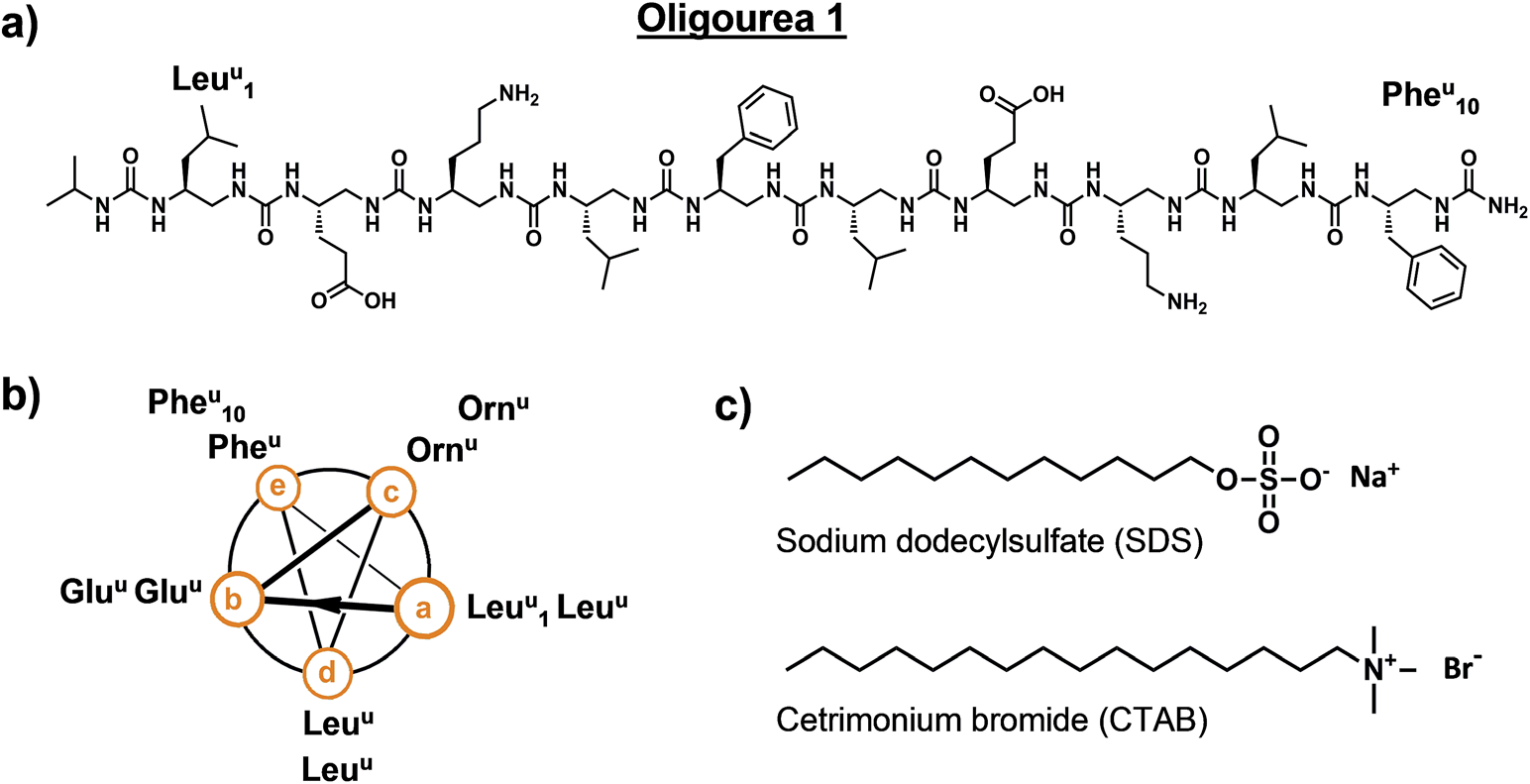
Details of oligourea **1** (a and b) and surfactants (c) used for co-crystallisation studies. Superscript ‘u’ denotes urea-type residue.

Oligourea **1** is an amphiphilic aliphatic oligourea 10 residues in length, bearing proteinogenic side-chains (Fig. 1a and b). This molecule was synthesised on solid support using azide succinimidyl carbamate building blocks following previously reported methods^43^ (for a full description of the chemistry methods see ESI†), yielding a pure and highly water-soluble molecule.‖
‖The solubility of oligourea **1** is >20 mg ml^–1^ in pure water. In order to understand the folding of this oligomer in an aqueous environment, we sought to crystallise oligourea **1** using standard protein crystallisation methods.

Crystallisation trials were performed at 20 °C in standard (aqueous) hanging drops composed of 0.5 μL of a solution of oligourea **1** at a concentration of 20 mg ml^–1^ (in pure water) plus an equal volume of crystallisation reagent. Several hundred unique crystallisation conditions from standard commercial sparse-matrix screens were used in an attempt to crystallise oligourea **1**, however, no crystals were obtained. We then employed focussed, specialised crystallisation screens (involving the systematic screening of salts and pH, for example), these too, however, proved unsuccessful in producing crystals. It was not until we serendipitously investigated surfactants as co-crystallising agents that we were able to grow single, well-formed crystals suitable for X-ray diffraction analysis. Oligourea **1** was finally crystallised using a crystallisation reagent composed of 0.5 M sodium chloride, 10 mM magnesium chloride, 100 mM sodium HEPES buffer (pH 7.0) and 10 mM of the cationic detergent cetrimonium bromide (CTAB) (Fig. 1c).

X-ray diffraction analysis of these crystals (using synchrotron radiation**
**Data were collected at SOLEIL synchrotron on beam line PROXIMA 1.) revealed good quality, high-resolution diffraction, indicative of well-ordered crystals. A full dataset was collected and processed to 1.44 Å, with the data belonging to space group *C*222_1_, with cell dimensions of *a* = 40.05 Å, *b* = 40.72 Å, *c* = 18.49 Å. The structure was solved by molecular replacement using a previously reported crystal structure of an oligourea obtained from crystals grown from an organic solvent crystallisation system^44^ (*i.e.* non-aqueous crystallisation). Matthews analysis indicated the asymmetric unit to be composed of a single copy of oligourea **1**, which was easily modelled into the initial electron density maps provided by the molecular replacement solution. Following this, however, a significant region of positive (*i.e.* unaccounted-for) electron density was evident in both 2*F*_o_ – *F*_c_ and *F*_o_ – *F*_c_ maps. A single molecule of CTAB was comfortably modelled into this electron density, resulting in a total of eight surfactant molecules and eight oligourea molecules per unit cell. The final model was refined using data to a resolution of 1.46 Å, with final *R*_work_ and *R*_free_ factors of 17.76 and 24.36%, respectively (Table 1). Full data collection and refinement details can be found in the ESI (Table S1†).

**Table 1:** Details of the four oligourea–surfactant co-crystal structures reported in this work

Structure	**1**	**2**	**3**	**4**
Space group	*C*222_1_	*P*2_1_2_1_2_1_	*C*222_1_	*C*222_1_
Resolution	1.46 Å	1.84 Å	1.49 Å	1.19 Å
*R*_work_ (%)	17.76	23.61	18.41	18.68
*R*_free_ (%)	24.36	26.89	25.12	22.03
Oligourea (+*Z* number)	**1** (8)	**1** (8)	**2** (8)	**3** (8)
Surfactant (+*Z* number)	CTAB^*a*^ (8)	SDS^*b*^ (8)	CTAB (8)	CTAB (8)
Surfactant vol.^*c*^ (as % of unit cell)	8.54	6.31	8.19	8.41
Solvent content^*d*^ (%) (oligourea only)	46.97	47.64	47.31	45.87
Solvent content^*d*^ (%) (oligourea + surfactant)	37.69	39.11	38.24	36.58
CCDC code	1050869	1050868	1050870	1050867

^*a*^CTAB, cetrimonium bromide.

^*b*^SDS, sodium dodecylsulfate.

^*c*^Surfactant volumes calculated using SURFNET.^45^

^*d*^Solvent content estimates using Matthews analysis^46^–^48^ of unit cells theoretically composed of either oligourea only or oligourea plus surfactant.

**Fig. 2:**
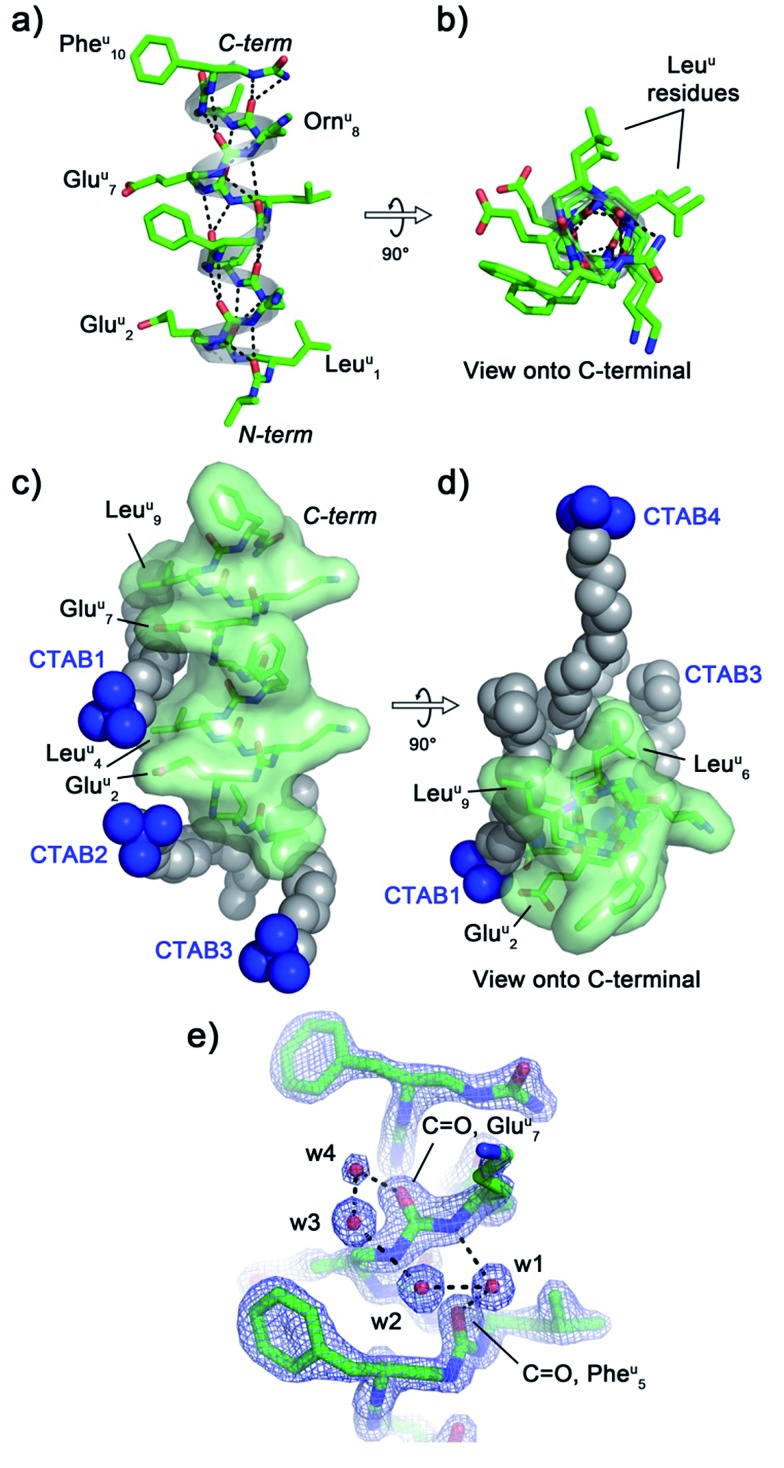
Crystal structure of oligourea **1** co-crystallised with the surfactant cetrimonium bromide (CTAB). (a and b) Oligourea **1** forms a canonical 2.5-helix when crystallised in the presence of CTAB. All intra-helical hydrogen bonds are present in this structure (black dashes). (c and d) Four distinct CTAB molecules (spheres) interact with a single oligourea **1** helix (green surface) *via* electrostatic and hydrophobic interactions. (e) Electron density for oligourea **1** surrounding a water bridge between the backbone carbonyl groups of Phe^u^5 and Glu^u^7 (2*F*_o_ – *F*_c_ map, σ level 2.1, resolution 1.46 Å).

The crystal structure of oligourea **1** reveals the foldamer to be fully helical, forming the expected canonical 2.5-helix typical of aliphatic oligoureas,^44^,^49^ with all possible intra-helical hydrogen bonds present (Fig. 2a and b, Table S2†). The 1 : 1 oligourea : surfactant ratio of the crystal structure results in the surfactant occupying a significant volume (8.54%, see Table 1) of the unit cell, with crystal packing contacts resulting in a total of four distinct surfactant molecules interacting with a single oligourea molecule (Fig. 2c and d).

Analysis of the helix geometry of the crystal structure of oligourea **1** reveals helical parameters almost identical to those of an exemplative canonical oligourea crystal structure derived from surfactant-free crystallisation conditions (Table 2), indicating that the surfactant does not negatively impact the secondary structure of this foldamer. Structural alignment of the CTAB-bound oligourea **1** structure reported here with this non-surfactant bound oligourea helix also reveals a high level of structural agreement (Cα r.m.s. deviation, 0.368 Å) (Table 3). In addition to apparently exerting no influence on foldamer secondary structure, the surfactant molecules do not appear to locally disrupt any regions of the foldamer either – all oligourea side-chains, backbone atoms and first hydration sphere are well-resolved in electron density (Fig. 2e), permitting details of the folding of this molecule in aqueous conditions to be understood at the atomic level. Of particular note is the observation of a chloride ion bound to the free N-terminal urea NH groups of the oligourea. A single chloride ion is hydrogen bonded to the NH groups of two oligourea helices arranged in head-to-head fashion, an arrangement analogous to that of natural chloride ion channels^50^ (ESI Fig. S1†). Oligoureas have shown promise as artificial anion receptors in non-aqueous conditions^51^,^52^ – the observation of a chloride ion bound to oligourea **1** in the crystal structure reported here suggests that the anion-binding ability of oligoureas could feasibly be extended to aqueous conditions, which would provide further possibilities for future application of these foldamers.

**Table 2:** Average helical parameters of oligourea–surfactant co-crystal structures. Parameters calculated using the HELANAL-plus server^53^

Crystal structure	Res/turn	Rise (Å)	Rise/turn (Å)	Radius (Å)	Twist/res (°)	Cα torsion (°)
**1** (CTAB)	2.49	2.05	5.10	2.77	144.54	97.38
**1** (SDS), chain A	2.50	2.05	5.13	2.78	143.96	96.51
**1** (SDS), chain B	2.50	2.04	5.10	2.79	143.94	96.26
**2** (CTAB)	2.49	2.07	5.15	2.76	144.41	97.75
**3** (CTAB)	2.49	2.06	5.13	2.77	144.61	97.72
Canonical oligourea helix^*a*^	2.48	2.03	5.03	2.70	145.41	99.71

^*a*^Crystal structure of a nona-urea oligourea helix derived from surfactant-free (organic solvent) crystallisation conditions (CCDC code 750017).^44^ Average helical parameter values for this structure were reported previously in Nelli *et al.*, 2013.^54^

**Table 3:** Structural alignment comparisons of surfactant-bound and surfactant-free oligourea crystal structures

Aligned structures	R.m.s.d.^*a*^ (Å)	No. of atoms
**1** (CTAB) *vs.* CCDC 750017	0.368	8 to 8 (Cα atoms)
**1** (SDS): chain A *vs.* chain B	0.703	115 to 115
**1** (CTAB) *vs.***1** (SDS, chain A)	0.393	115 to 115
**1** (CTAB) *vs.***1** (SDS, chain B)	0.805	115 to 115
**2** (CTAB) *vs.* CCDC 750017	0.396	8 to 8 (Cα atoms)
**3** (CTAB) *vs.* CCDC 750017	0.426	8 to 8 (Cα atoms)

^*a*^R.m.s.d., root-mean-square deviation. R.m.s.d. values determined from structural alignments performed in PyMOL.^55^

CTAB molecules interact with hydrophobic regions of the oligourea, such as the leucine-type side-chains of the urea-leucine residues (Leu^u^ [superscript ‘u’ denotes urea-residue]), as well as with charged regions, such as the glutamate-type side-chains of the urea-glutamate residues (Glu^u^), effectively ‘sticking’ foldamer molecules together in the crystal lattice (Fig. 2c and d). For example, the charged trimethylammonium head of ‘CTAB **1**’ (as labelled in Fig. 2c and d) is situated within electrostatic-bonding distance of the charged glutamate-type side-chains of residues Glu^u^2 and Glu^u^7. The alkyl chain of this CTAB molecule then associates through hydrophobic interactions with the leucine-type side-chains of residues Leu^u^4 and Leu^u^9 of the same oligourea molecule, with the terminus of the CTAB alkyl chain interacting with two additional molecules of oligourea **1** in the crystal lattice (ESI Fig. S2†). In addition, it appears that the CTAB molecules occupy regions of the lattice otherwise filled with disordered bulk solvent. Indeed, based on Matthews estimates, a significant percentage of solvent (almost 10%) is replaced with the more structurally ordered surfactant molecules (Table 1), which would be expected to contribute significantly to crystal packing interactions and improve the overall ordering of the crystal. Thus it seems that the CTAB surfactant promotes crystal growth through two routes: (1) by bridging inter-foldamer contacts in the crystal lattice, thereby acting as ‘molecular glue’ and (2) by displacing disordered bulk solvent with better-ordered lattice components (*i.e.* the surfactant).

In order to investigate whether alternative surfactants could be employed in a similar manner – *i.e.* to promote crystal growth by acting as ‘molecular glue’ – we attempted to crystallise oligourea **1** in the presence of the common anionic surfactant sodium dodecylsulfate (SDS). Crystallisation experiments similar to those described above – but with SDS in place of CTAB – yielded good-quality crystals suitable for X-ray diffraction analysis. A 1.84 Å resolution dataset was collected for a crystal of oligourea **1** grown in the presence of SDS. The data were processed as above, and were indexed and integrated in space group *P*2_1_2_1_2_1_, with cell dimensions (in Å) of: *a* = 18.49, *b* = 40.26, *c* = 41.02. The structure was solved by molecular replacement, with two copies of oligourea **1** in the asymmetric unit. Towards the end of the refinement process, two molecules of SDS were modelled into appropriate residual electron density, resulting in a 1 : 1 surfactant : oligourea ratio, analogous to the equivalent CTAB structure above. The final model was refined to a resolution of 1.84 Å, with *R*_work_ and *R*_free_ factors of 23.61 and 26.89%, respectively (see Tables 1 and S1†).

**Fig. 3:**
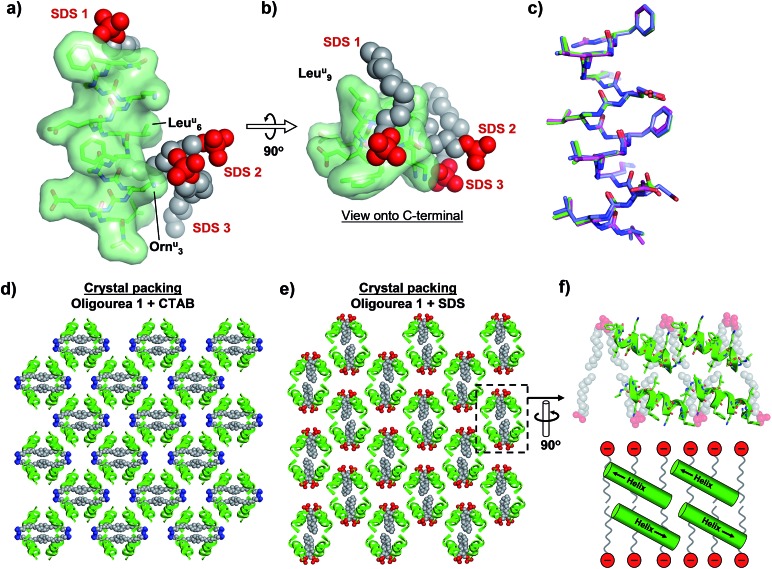
(a and b) Crystal structure of oligourea **1** co-crystallised with sodium dodecylsulfate (SDS). (c) Structural alignment of crystal structures of oligourea **1** bound by cetrimonium bromide (CTAB) (green carbons) and SDS (magenta carbons, chain A; blue carbons, chain B). R.m.s.d. values are shown in Table 3. (d and e) Crystal packing of oligourea **1** co-crystallised in the presence of CTAB (d) or SDS (e). Lattices are in the same orientation (relative to the oligourea of the asymmetric unit). (f) Cross-section of oligourea **1**-SDS lattice showing ‘bilayer-like’ arrangement of SDS in the crystal lattice (top, crystal structure; bottom, schematic).

As with the CTAB–oligourea **1** co-crystal structure described above (structure **1**), the oligourea molecules of the SDS complex (structure **2**) are fully helical, with no deviations from the expected helical geometry and with all expected intra-helical hydrogen bonds present (Fig. 3a and b, Tables 2 and S2†). Structural alignment of the two crystallographically unique oligourea chains of the SDS complex reveals a high level of correlation, with an r.m.s. deviation of 0.703 Å (for 115 *vs.* 115 atoms) (Fig. 3c and Table 3). More significantly, structural alignment of the oligourea **1** helices from the CTAB and SDS co-crystal structures also reveals a high level of similarity – with r.m.s. deviation values of 0.393 and 0.805 Å for alignments of structure **1***vs.* chain A and chain B of structure **2**, respectively – indicating that the nature of the co-crystallising surfactant does not appear to impact the oligourea folding or geometry (Fig. 3c and Table 3). Interestingly, the SDS molecules do not occupy the same positions in the crystal lattice (relative to oligourea **1**) as the CTAB molecules in the equivalent structure reported above, but instead are re-orientated by 90° (Fig. 3d and e) – seemingly in order to maximise electrostatic contacts between the surfactant sulfate groups and the positively charged ornithine-type (Orn^u^) side-chains of the oligourea. Despite the SDS molecules occupying different positions in the crystal lattice (compared to CTAB in structure **1**), the anionic SDS molecules play a similar role to the CTAB in crystal packing, involving: (1) the intermolecular bridging of oligourea molecules and (2) the replacement of disordered bulk solvent (Table 1). This suggests that certain surfactants possess a degree of intrinsic versatility as opportunistic components of aqueous crystal lattices, making these molecules potentially useful crystallogenesis-promoting tools.

In addition, the re-orientation of the SDS molecules in the crystal lattice generates a curious arrangement comparable, to some degree, to a phospholipid bilayer of a cell membrane (Fig. 3f). Although the foldamers reported here have no known anti-bacterial activity, there is considerable interest in the development of peptides and peptidomimetic molecules (including oligoureas^56^,^57^) as anti-bacterial agents.^58^ One mode of action of some such peptides is thought to involve peptide-membrane interactions, however, high-resolution structural details of anti-bacterial peptide-membrane interactions are understandably challenging to obtain. Thus, our results suggest that it may be worthwhile for those engaged in such research to consider the use of anionic surfactants as co-crystallising reagents (in conjunction with the peptide-of-interest) as a means to generate a ‘model-membrane’ in a crystal lattice, and thereby permit potentially valuable structural information to be obtained.

**Fig. 4:**
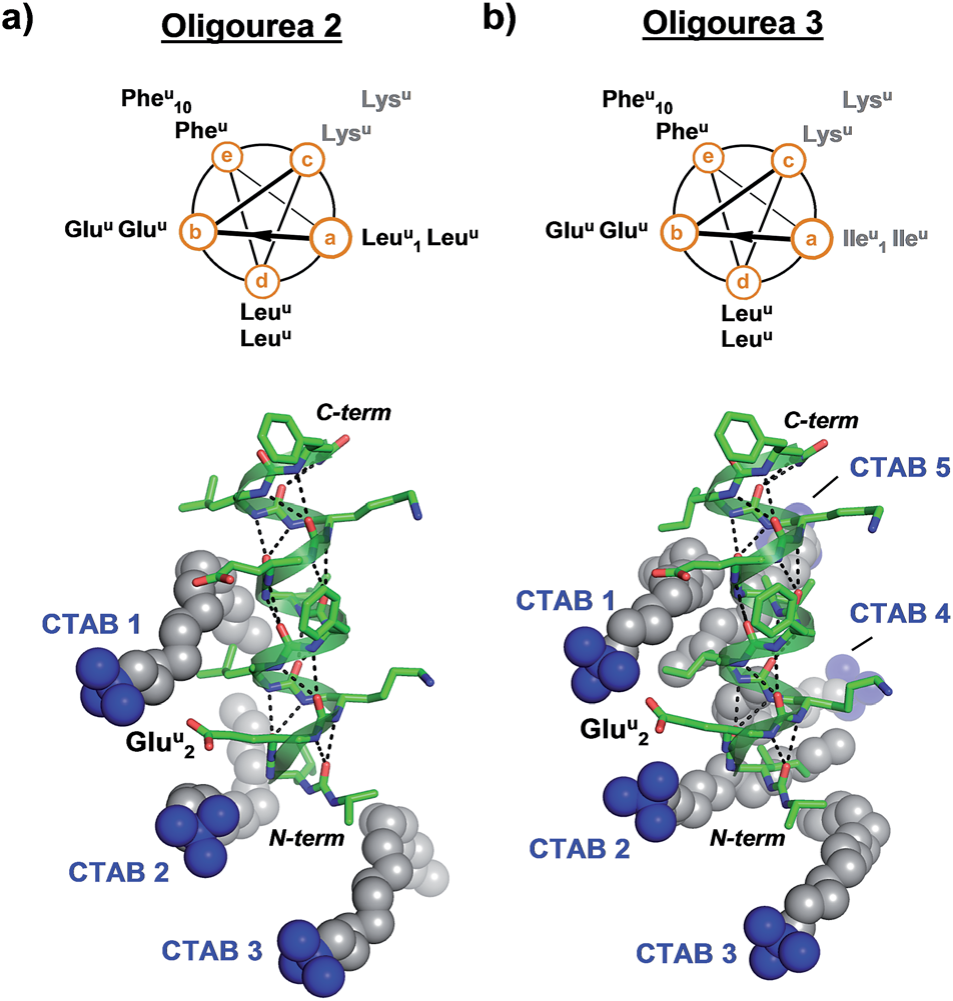
Helical-wheel diagrams and co-crystal structures of oligoureas **2** (a) and **3** (b) bound by cetrimonium bromide (CTAB). Grey labels on helical-wheels correspond to residues modified relative to oligourea **1**. The crystal structures are in the same orientation as oligourea **1** in Fig. 2c and were refined to final resolutions of 1.49 Å and 1.19 Å for oligoureas **2** and **3**, respectively.

In order to test whether the method of using surfactants to promote crystal growth could be extended to additional difficult-to-crystallise foldamers, we performed crystallisation trials in the presence and absence of surfactants for two additional oligourea molecules – oligoureas **2** and **3** (Fig. 4). These foldamers are analogues of oligourea **1** – oligourea **2** contains lysine-type urea residues (Lys^u^) in place of the ornithine-type urea residues (Orn^u^), with oligourea **3** bearing this same Orn^u^ to Lys^u^ replacement, in addition to all leucine-type (Leu^u^) urea residues being replaced with isoleucine-type (Ile^u^) urea residues (Fig. 4). As with oligourea **1**, sparse-matrix crystallisation screening of oligoureas **2** and **3** failed to yield crystals (full details of crystallisation experiments can be found in the ESI†), however, use of crystallisation reagents containing CTAB yielded well-ordered good quality single crystals for both foldamers. Diffraction data collected for crystals of oligoureas **2** and **3** resulted in successful structure determination for both foldamers. Both structures belong to space group *C*222_1_, with resolutions of the final refined models of 1.49 Å and 1.19 Å for oligoureas **2** and **3**, respectively (further crystallographic details can be found in Tables 1 and S1†). As expected, the crystal structures reveal oligoureas **2** and **3** to form well-folded 2.5-helices, with average helical metrics and folding characteristics almost identical to those of oligourea **1** (Tables 2 and 3 and S2†). Importantly, the crystal structures of oligoureas **2** and **3** both reveal the presence of well-ordered CTAB molecules playing key roles in forging crystal packing contacts (Fig. 4). These additional high-resolution crystal structures thus provide further evidence in support of the proposition that certain surfactant molecules can exert a dramatic and positive influence on the outcome of aqueous foldamer crystallisation endeavours.

## Conclusions

The amphiphilic nature of surfactants makes these molecules well-suited for use as ‘molecular glue’ in crystal lattices, as they have the ability to interact with both hydrophilic and hydrophobic moieties. In the examples reported here, the surfactants appear to aid crystal growth in two ways: (1) by linking foldamer molecules in the crystal lattice, and (2) by replacing disordered bulk solvent with ordered crystalline components. We have shown here that both anionic and cationic surfactants can promote the crystallogenesis of fully water-soluble oligourea foldamers which had previously proven to be resistant to crystallisation, permitting high resolution crystal structures to be determined with resolutions of up to 1.19 Å. The considerable level of structural information provided by these high-resolution crystal structures – made possible only through the inclusion of surfactants as co-crystallising reagents – suggests that the use of surfactants as promoters of crystallogenesis may be well worth considering for those engaged in (or embarking upon) challenging aqueous crystallographic studies of water-soluble foldamers (or peptides). Although it should of course be noted that, as the findings described herein are confined to a single class of foldamer, further investigations will be required in order to determine whether surfactants are indeed able to aid the crystallisation of additional classes of foldamers (such as β-peptides).

## Supplementary Material

Supplementary information

Crystal structure data
