# Supplementary material for: Surfactant-facilitated crystallisation of water-soluble foldamers
Source: Chem Sci. 2016 Feb 10;7(5):3377–83. doi: 10.1039/c6sc00090h (PMC6006954; doi:10.1039/c6sc00090h)
Supplement: Supplementary file 1 [file SC-007-C6SC00090H-s001.pdf]

Electronic Supplementary Material (ESI) for Chemical Science.
This journal is © The Royal Society of Chemistry 2016

Surfactant-facilitated crystallisation of water-soluble foldamers.

Gavin W. Collie, Karolina Pulka-Ziach and Gilles Guichard.

Supplementary information

Supplementary figures

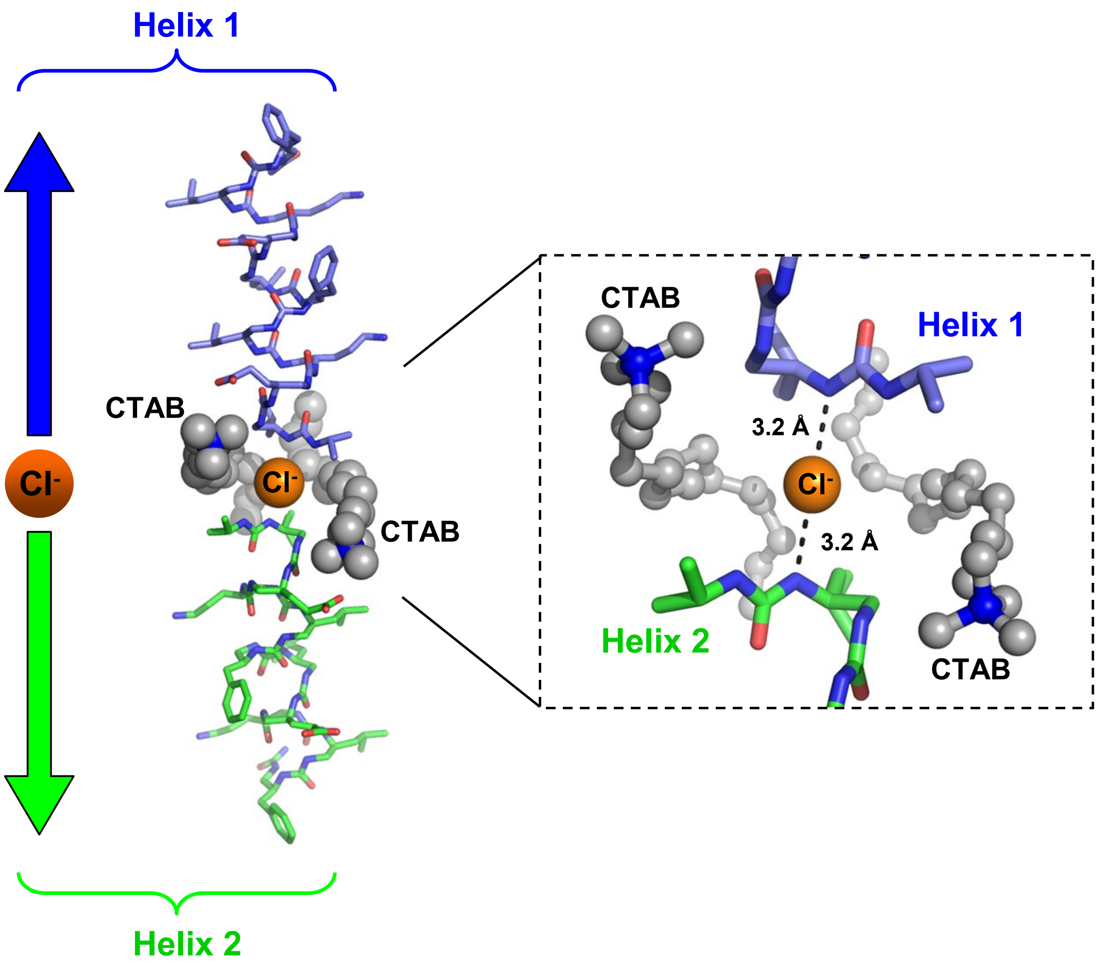

Figure S1. Co-crystal structure of oligourea **1** plus CTAB (cetrimonium bromide) showing details of chloride binding. One chloride ion (orange sphere) can be seen coordinated between the N-terminal free NH groups of two separate oligourea **1** helices (distinguished by carbon colour [blue or green]) in the crystal lattice.

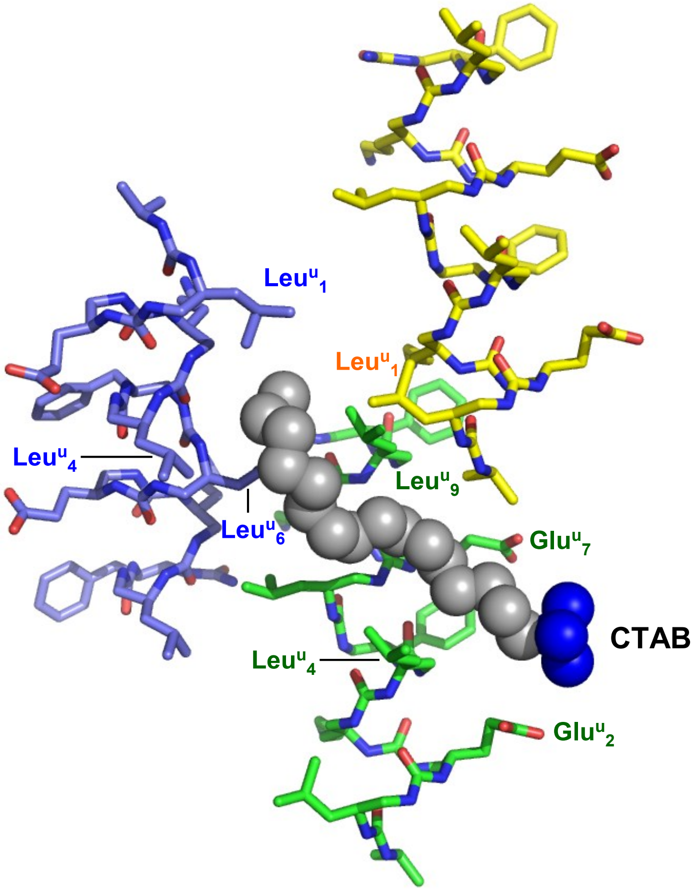

Figure S2. Co-crystal structure of oligourea **1** plus CTAB (cetrimonium bromide) showing the interactions of a single CTAB molecule with multiple oligourea **1** molecules in the crystal lattice. Distinct oligourea **1** molecules are distinguished by carbon colour. The CTAB molecule shown here corresponds to 'CTAB 1' in figure 2. The key residues within range of CTAB suitable for electrostatic or hydrophobic interactions are labelled.

Supplementary methods

Chemistry

Oligoureas **1-3** were synthesised on solid support using azide protected building blocks (Scheme 1), which were synthesised starting from *N*-Boc or *N*-Cbz protected (L) amino acids following previously reported procedures[1]. The synthesis of all monomer building blocks except the azide-protected isoleucine-type building block has been reported previously[1,2]. Details of the azide-protected isoleucine-type building block are reported below, followed by a description of the solid phase synthesis of oligoureas **1-3**.

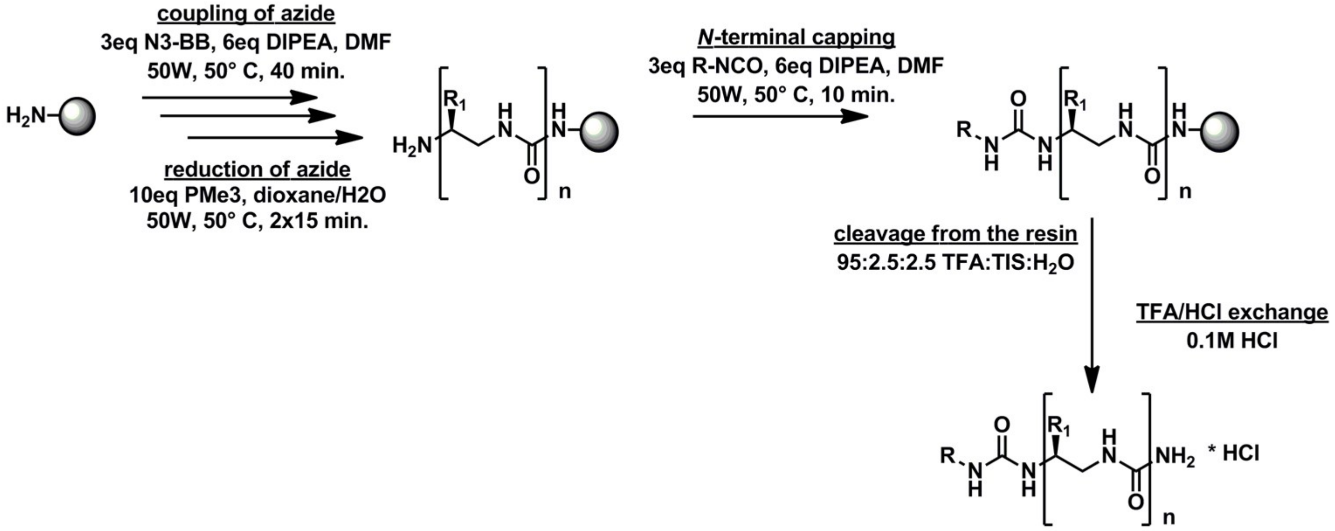

Scheme 1. General scheme of solid phase synthesis (SPS) of oligoureas under microwave irradiation.

Azide protected building block synthesis for oligourea synthesis on solid support

2,5-dioxopyrrolidin-1-yl ((*2S,3S*)-2-azido-3-methylpentyl)carbamate (N_3-Ile^u-OSu building block)

The pure product was obtained as a white solid, with an overall yield 32 % after 6 steps. ^{1}H NMR ($CDCl_3$, 300 MHz) δ 5.52 (bs, 1H), 3.62 – 3.48 (m, 2H), 3.21 – 3.07 (m, 1H), 2.87 (s, 4H), 1.80 – 1.67 (m, 1H), 1.64-1.51 (m, 1H), 1.39-1.22 (m, 1H), 1.01 (d, *J* = 6.8 Hz, 1H), 0.98 (t, *J* = 7.4 Hz, 3H); ^{13}C NMR ($CDCl_3$, 75 MHz) δ 169.84, 151.61, 66.62, 42.96, 37.19, 25.48, 15.06, 11.38.

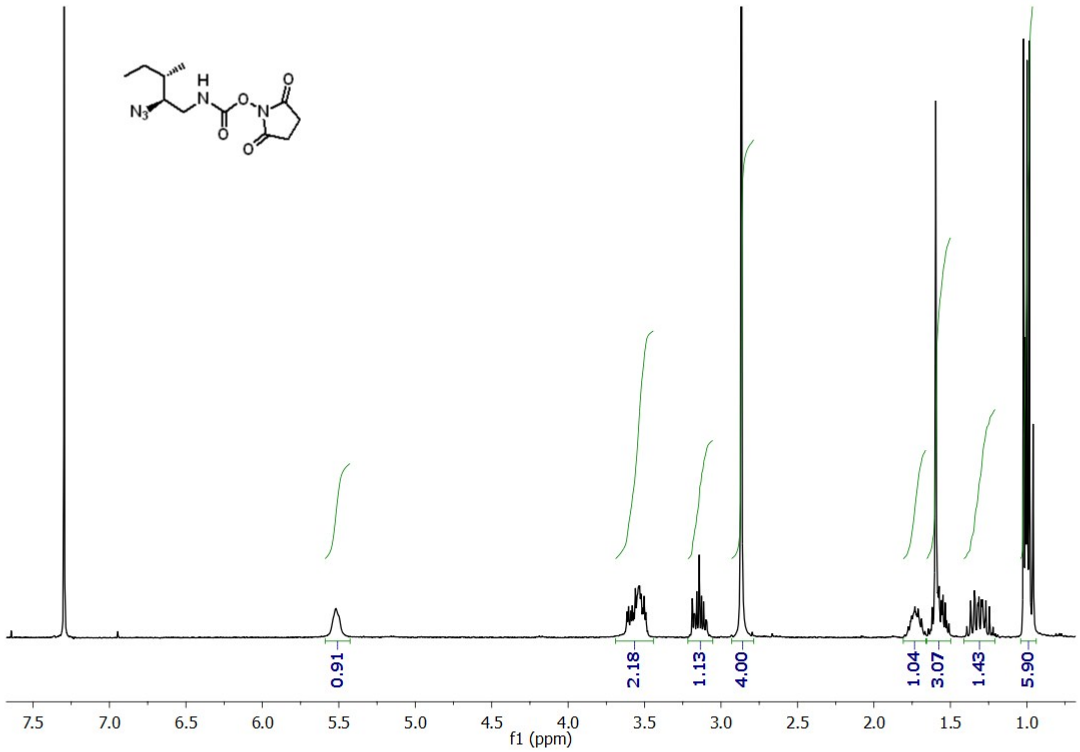

Figure S3. ^{1}H NMR of 2,5-dioxopyrrolidin-1-yl ((*2S,3S*)-2-azido-3-methylpentyl)carbamate (N_3-Ile^{u}-OSu).

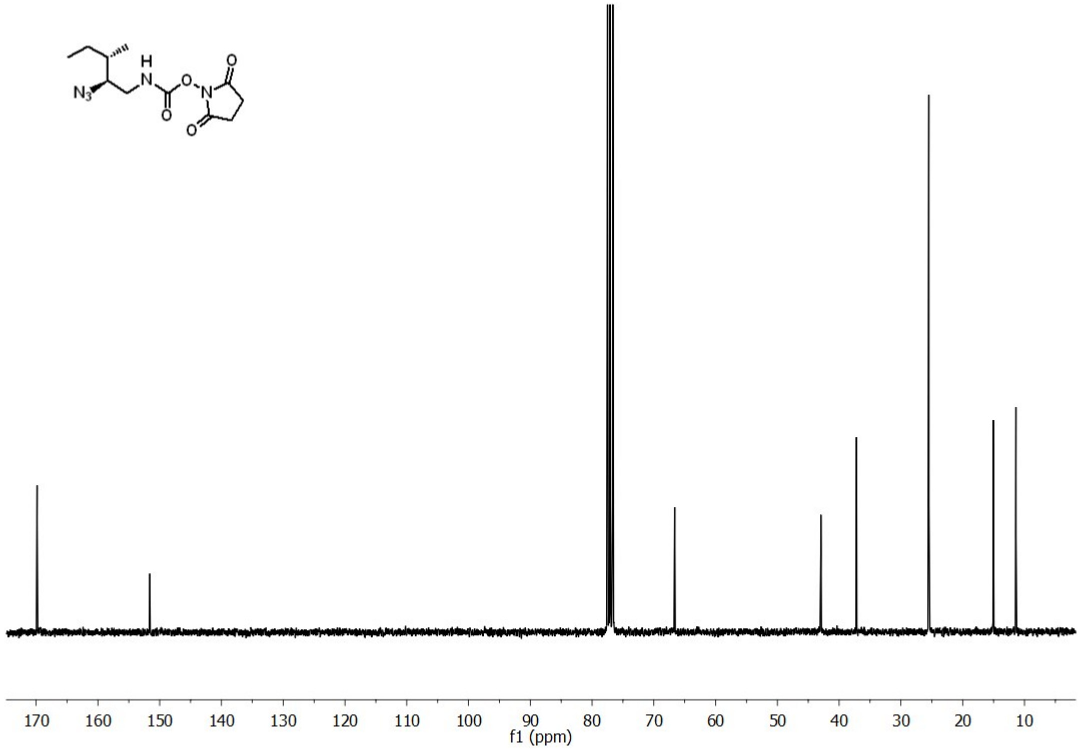

Figure S4. ^{13}C NMR of 2,5-dioxopyrrolidin-1-yl ((*2S,3S*)-2-azido-3-methylpentyl)carbamate (N_3-Ile^{u}-OSu).

Solid phase oligourea synthesis under microwave conditions.

Oligoureas were synthesized as previously reported[1] in polypropylene SPE tubes (CEM) using microwave assisted solid phase synthesis (CEM Discover) on NovaPeg Rink Amide resin (Novabiochem) using 0.017 mmol scales. The syntheses were monitored using the chloranil test[3]. Unless stated otherwise, the reagents were purchased from Sigma Aldrich.

The resin (50 mg, 17 µmol) was swelled with DMF (1.5 mL) for 2-24 h. Activated N_3-building block (51 µmol, 3eq relative to the resin loading) was dissolved in DMF (1.5 mL) and was added to the reaction vessel, followed by DIPEA (102 µmol, 6 eq relative to the resin loading). The vessel was then placed inside the microwave reactor and irradiated (50 W, 50 °C, 40 min.). All microwave experiments were conducted at atmospheric pressure. The temperature was maintained by modulation of power and controlled with a fiber optic sensor. After 40 min., the resin was filtered and washed with DMF (4 x 2 mL). Reduction of the azide group was performed using a mixture of 1,4-dioxane:H_2O (7:3 v/v). Before the reaction, the resin was washed with this solvent mixture. The Staudinger reaction was then performed under microwave irradiation (50 W, 50 °C, 15 min. x 2) in 1,4-dioxane:H_2O (1.5 mL), with 1 M PMe_3 solution in THF (0.17 mL, 170 µmol, 10 eq relative to the resin loading) as the reducing agent. After the reaction, the resin was filtered and washed with 1,4-dioxane:H_2O (1 x 2 mL) and DMF (4 x 2 mL). If necessary the coupling or reduction steps were repeated. *N*-terminal capping was carried out with isopropyl isocyanate (51 µmol, 3eq relative to the resin loading) in the presence of DIPEA (102 µmol, 6 eq relative to the resin loading) under microwave irradiation (50 W, 50 °C, 10 min.). When the synthesis was finished, the resin was transferred into a syringe with a frit, washed with DMF (5 x 2 mL), DCM (5 x 2 mL), MeOH (5 x 2 mL), Et_2O (5 x 2mL) and dried in a desiccator.

The compounds were cleaved from the resin with a mixture of 95:2.5:2.5 TFA:TIS:H_2O (1 mL) for 3.5 h. Following this, the resin was filtered and washed with TFA (1 x 1 mL), ACN (2 x 1 mL), MeOH (2 x 1 mL), ACN (1 x 1 mL). All the filtrates were combined and the solvents were evaporated to dryness. The crude products were precipitated with cold Et_2O as TFA salts and purified with the appropriate gradient by semipreparative HPLC (Dionex Ultimate 3000, column Macherey-Nagel Nucleodur 100-16 C18 ec, 10 x 250, solvents acetonitrile/water 0.1 % TFA, 4 ml/min). Analytic HPLC characterizations were performed on Macherey-Nagel column, Nucleodur cc 70/4 100-3 C18 ec, 4.6 x 100, solvents acetonitrile/water 0.1 % TFA, 1 ml/min. The compounds were freeze-dried and TFA was exchanged with HCl by repeated lyophilisation using 0.1 M HCl.

Oligourea 1

iPr-NHCO-Leu^u-Glu^u-Orn^u-Leu^u-Phe^u-Leu^u-Glu^u-Orn^u-Leu^u-Phe^u-NH$_2$

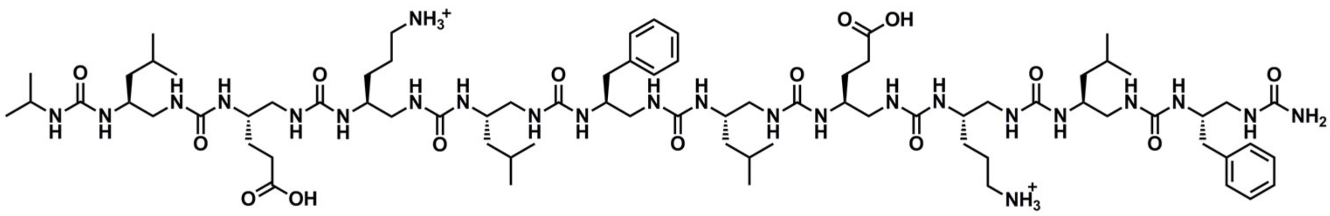

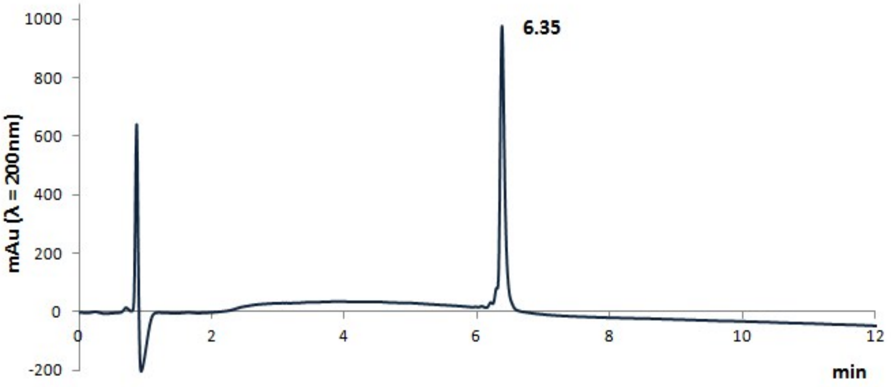

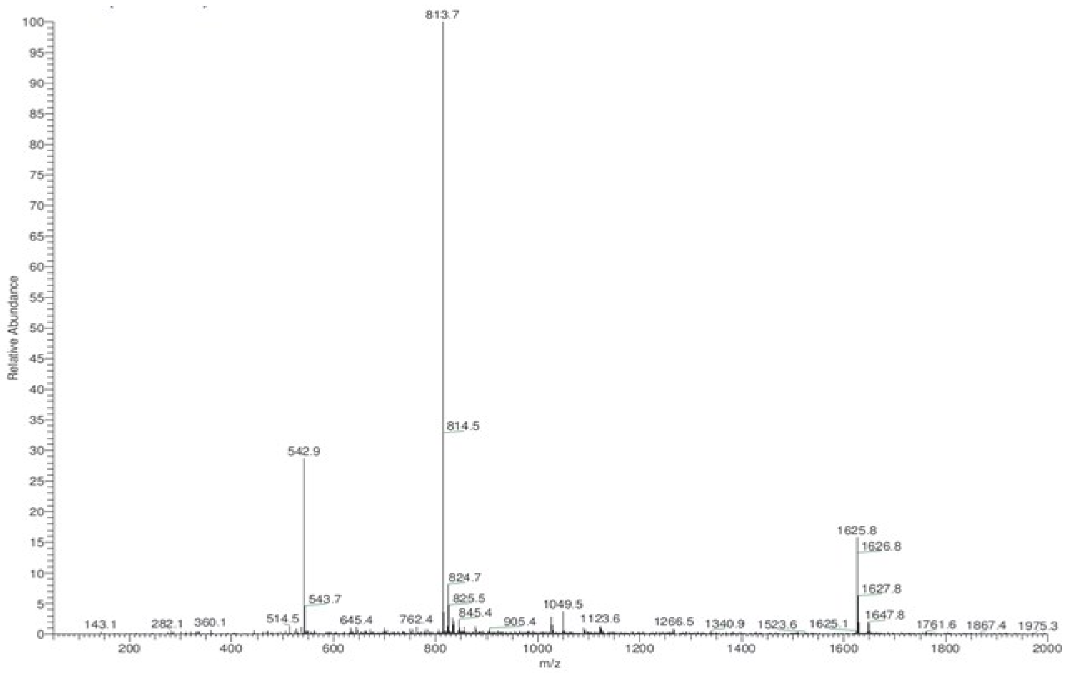

Figure S5. Characterisation of oligourea **1**.
Top: chemical structure of oligourea **1**. Middle: HPLC, R_t= 6.35 min (30-55 %/5 min., 55-60 %/5 min., 60-100 %/2 min CH_3CN 0.1 % TFA in H_2O 0.1 % TFA, C18, λ=200nm). Bottom: ESI-MS (ESI+) *m/z*: 542.9 $[M+3H]^{3+}$, 813.7 $[M+2H]^{2+}$, 824.7 $[M+Na+H]^{2+}$, 1625.8 $[M+H]^{+}$, 1647.8 $[M+Na]^{+}$.

Oligourea 2

iPr-NHCO-Leu^u-Glu^u-Lys^u-Leu^u-Phe^u-Leu^u-Glu^u-Lys^u-Leu^u-Phe^u-NH$_2$

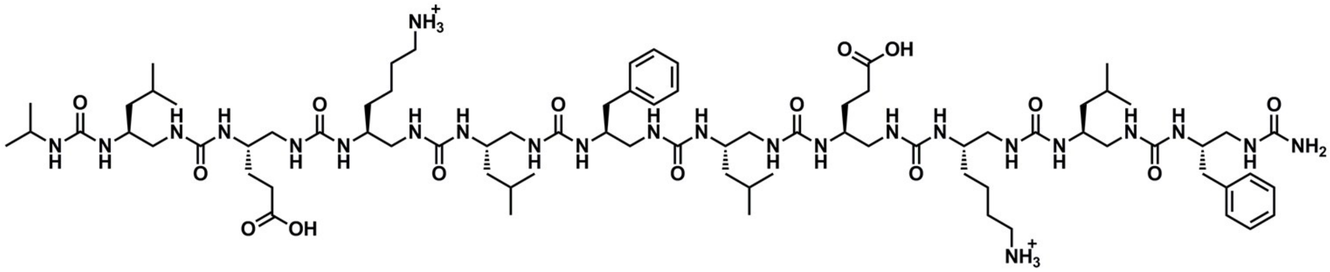

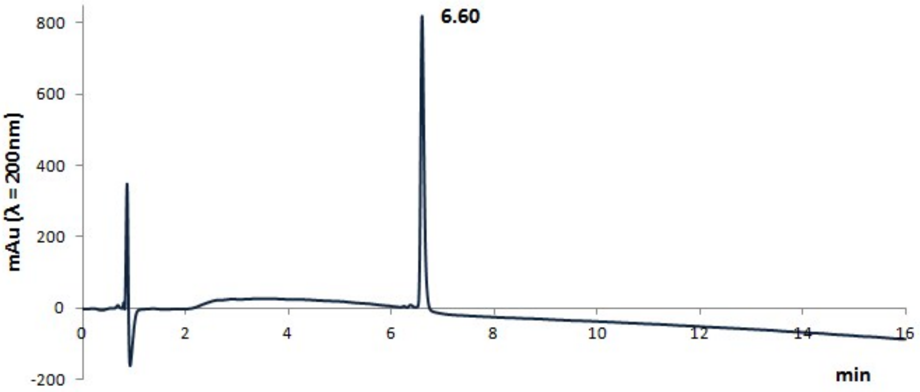

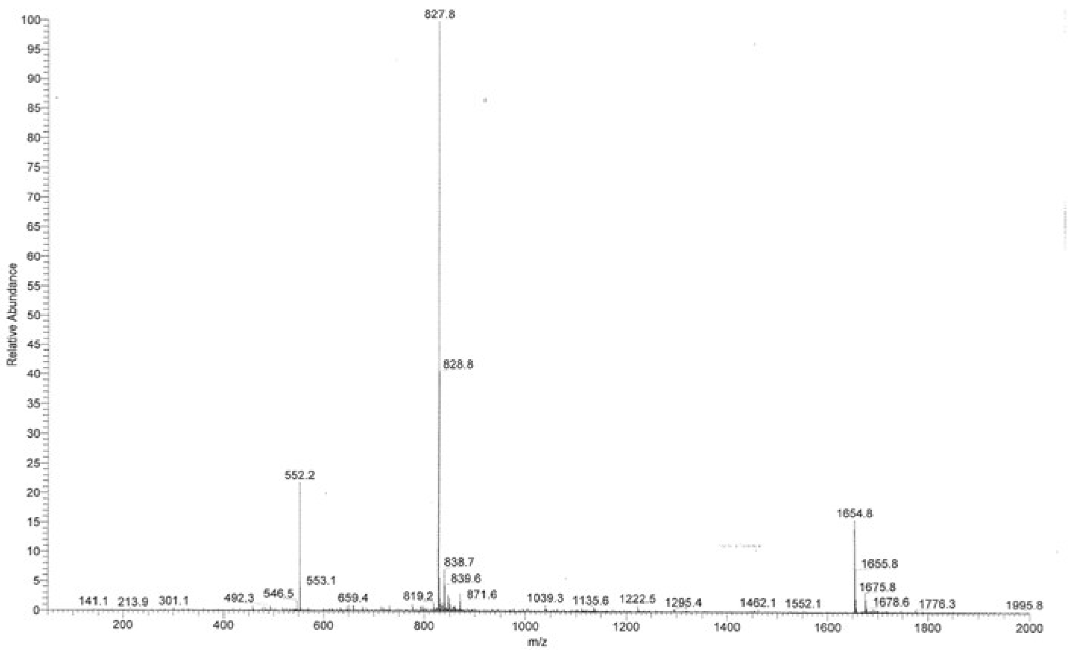

Figure S6. Characterisation of oligourea **2**.
Top: chemical structure of oligourea **2**. Middle: HPLC, R_t= 6.60 min (30-55 %/5 min., 55-65 %/10 min., 65-100 %/1 min CH_3CN 0.1 % TFA in H_2O 0.1 % TFA, C18, λ=200nm). Bottom: ESI-MS (ESI+) *m/z*: 552.2 $[M+3H]^{3+}$, 827.8 $[M+2H]^{2+}$, 838.7 $[M+Na+H]^{2+}$, 1654.8 $[M+H]^{+}$.

Oligourea 3

iPr-NHCO-Ile^u^-Glu^u^-Lys^u^-Ile^u^-Phe^u^-Ile^u^-Glu^u^-Lys^u^-Ile^u^-Phe^u^-NH$_2$

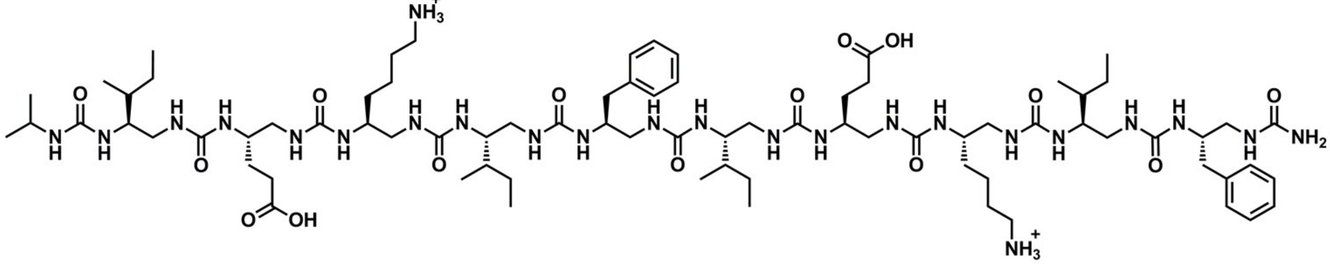

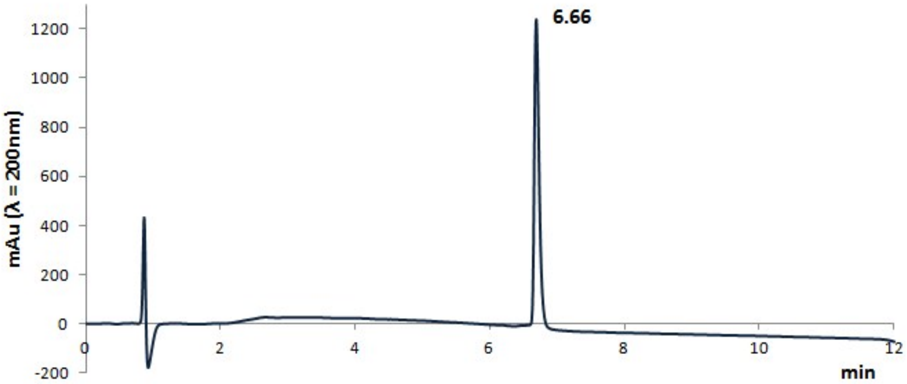

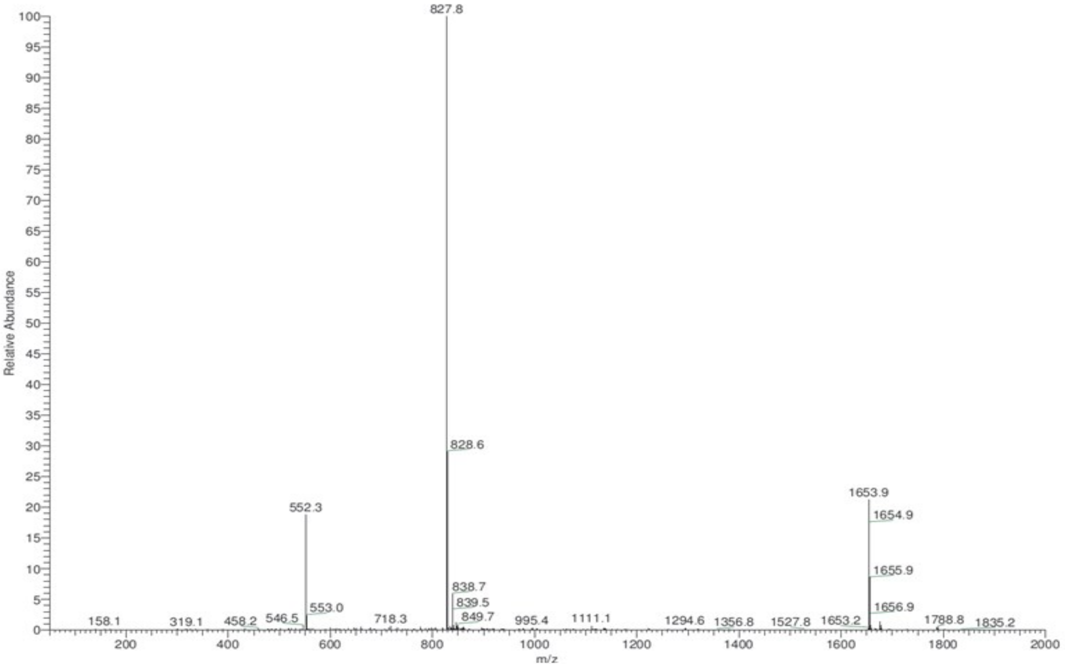

Figure S7. Characterisation of oligourea **3**.
Top: chemical structure of oligourea **3**. Middle: HPLC, R_t= 6.66 min (30-55 %/5 min., 55-60 %/5min., 60-100 %/2 min CH_3CN 0.1 % TFA in H_2O 0.1 % TFA, C18, λ=200nm). Bottom: ESI-MS (ESI+) *m/z*: 552.3 $[M+3H]^{3+}$, 827.8 $[M+2H]^{2+}$, 838.7 $[M+Na+H]^{2+}$, 1654.9 $[M+H]^{+}$.

Crystallography

Crystallisation

For crystallisation trials, oligoureas **1-3** were dissolved in double-distilled H_2O to final concentrations of 20 mg/ml. Crystallisation trials were performed in standard aqueous hanging drops at 20 ^oC composed of 0.5 µL of oligourea solution plus an equal volume of crystallisation reagent. Initially, standard commercial sparse-matrix crystallisation screens were used in an attempt to obtain suitable crystals for X-ray diffraction analysis. These attempts proved unsuccessful, and were followed by use of more focussed and specialised screens (involving the systematic screening of salts and pH, for example). These crystallisation attempts also proved to be unsuccessful, and it was not until we investigated surfactants as co-crystallising agents that we were able to grow single, well-formed crystals suitable for X-ray diffraction analysis. (It should be noted that these foldamers are fully soluble in pure water, and that the surfactants were not needed as solubilising agents.) Crystals of oligoureas **1-3** were grown using crystallisation reagents composed of 0.02 – 0.5 M NaCl, 10 – 12.5 mM surfactant (cetrimonium bromide [CTAB] or sodium dodecylsulfate [SDS]), 10 mM $MgCl_2$ and 0.1 M sodium HEPES buffer (pH 7.5) (in the case of oligourea **1** plus CTAB only). See Figure S8 for representative pictures of crystals. Specific details of crystallisation conditions are shown in Table S1.

Data collection, structure solution and refinement

Single crystals of oligoureas **1-3** grown in the presence of surfactant were cryo-protected for 1 – 2 minutes in solutions composed of 67 % of the concentration of mother liquor supplemented with 25 % glycerol before being flash-frozen in liquid nitrogen. Diffraction data were collected at 100 K on beam line PROXIMA 1 at SOLEIL synchrotron. The diffraction resolutions were in the range of 1.19 – 1.84 Å (Table S1). Diffraction data were processed in XDS[4] and Scala[5,6]. Crystals grown in the presence of CTAB belong to space group $C\ 222_1$, with crystals grown in the presence of SDS belonging to space group $P\ 2_12_12_1$. All four structures were solved by molecular replacement using Phaser[7], using a modified crystal structure of a nona-urea oligourea foldamer as a search model (CCDC code 750017[8]). Model building and restrained refinement were preformed in Coot[9] and REFMAC5[10], respectively. Geometric restraints were generated using the PRODRG server[11]. Details of unit cell dimensions, resolutions and final refinement statistics can be found in Table S1. Atomic coordinates and structure factors for all four crystal structures have been deposited in the Cambridge Crystallographic Data Centre (accession codes in Table S1). Please note: CCDC entries for these structures also contain the original .pdb files, which have been embedded within the deposited .cif files.

Additional

Helical analysis of the oligourea crystal structures was performed using the HELANAL-plus server[12,13]. Additional conformational and geometric analyses were performed using PyMOL[14]. Surfactant and oligourea volumes were calculated using SURFNET[15]. All figures were prepared in PyMOL.

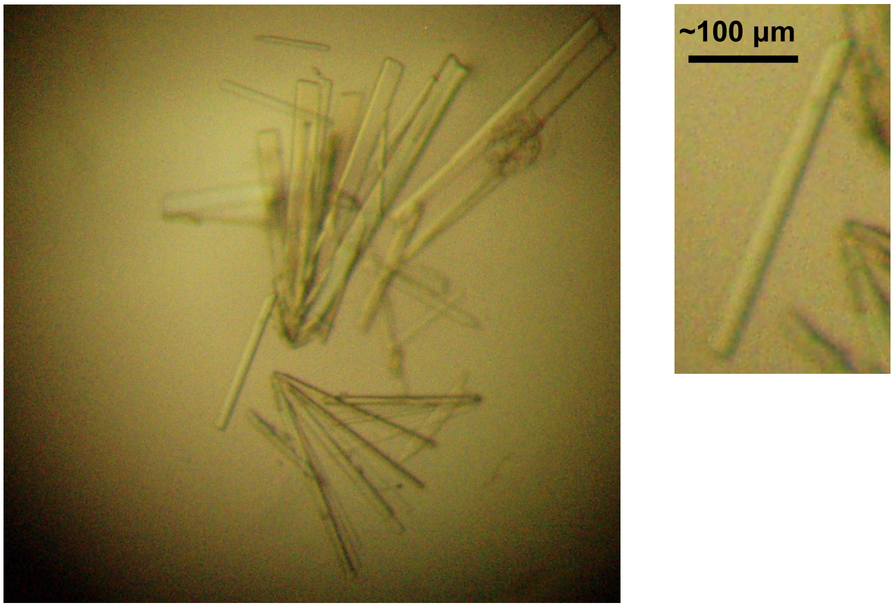

Figure S8. Hanging drop containing crystals of oligourea **3**. The hanging drop is composed of 0.5 µL of a 20 mg/ml solution of oligourea **3** plus an equal volume of a crystallisation reagent composed of 0.25 M NaCl, 12.5 mM cetrimonium bromide (CTAB) and 10 mM $MgCl_2$. These crystals appeared after 2 days and were left to grow for approximately 10 days before data collection. The crystals shown here are representative of all co-crystal complexes described in this work. Inset: magnified crystal from the left panel.

Table S1. Crystallographic data collection and refinement statistics for oligourea-surfactant crystal structures.

	**Structure 1**	**Structure 2**	**Structure 3**	**Structure 4**
Oligourea (sequence)	Oligourea **1** $L^uE^uO^uL^uF^uL^uE^uO^uL^uF^u$	Oligourea **1** $L^uE^uO^uL^uF^uL^uE^uO^uL^uF^u$	Oligourea **2** $L^uE^uK^uL^uF^uL^uE^uK^uL^uF^u$	Oligourea **3** $I^uE^uK^uI^uF^uI^uE^uK^uI^uF^u$
Crystallisation conditions	0.5 M NaCl<br>10 mM CTAB[a]<br>10 mM $MgCl_2$<br>0.1 M Na-HEPES pH 7.0	20 mM NaCl<br>12 mM SDS[b]<br>10 mM $MgCl_2$	0.5 M NaCl<br>10 mM CTAB<br>10 mM $MgCl_2$	0.25 M NaCl<br>12.5 mM CTAB<br>10 mM $MgCl_2$
Data Collection				
Space group	*C* 222_1	*P* $2_12_12_1$	*C* 222_1	*C* 222_1
Unit cell				
a, b, c (Å)	40.05, 40.72, 18.49	18.49, 40.26, 41.02	40.17, 40.85, 18.80	39.29, 40.15, 19.05
α, β, γ (°)	90.0, 90.0, 90.0	90.0, 90.0, 90.0	90.0, 90.0, 90.0	90.0, 90.0, 90.0
Unit cell volume	30154.16	30535.59	30849.76	30051.25
Oligourea Z number	8	8	8	8
Surfactant (+ Z number)	CTAB (8)	SDS (8)	CTAB (8)	CTAB (8)
Resolution (Å)	28.55 – 1.44 (1.53 – 1.44)[c]	41.02 – 1.84 (1.95 – 1.84)	28.64 – 1.49 (1.58 – 1.49)	28.08 – 1.19 (1.26 – 1.19)
R_{meas}[d] (%)	3.7 (51.7)	5.3 (59.6)	3.7 (41.1)	6.1 (30.3)
I / σ	21.92 (3.35)	20.73 (3.66)	26.52 (4.63)	21.51 (5.96)
Completeness (%)	99.3 (97.8)	97.8 (96.0)	99.7 (98.2)	98.7 (93.9)
Reflections (total)	17107	19162	21463	31502
Reflections (unique)	2921	2854	2707	5027
Redundancy	5.9	6.7	7.9	6.3
Refinement				
Resolution (Å)	8.00 – 1.46	28.74 – 1.84	28.64 – 1.49	28.08 – 1.19
R_{work} / R_{free} (%)	17.76 / 24.36	23.61 / 26.89	18.41 / 25.12	18.68 / 22.03
No. of atoms	153	276	157	159
Ions	0.5	1	2.5	2.5
Waters	17	11	17	19
Overall B-factor ($Å^2$)	36.40	26.91	34.37	22.03
R.m.s. deviations				
Bond lengths (Å)	0.012	0.013	0.010	0.020
Bond angles (°)	1.871	1.7318	1.757	1.942
CCDC code	1050869	1050868	1050870	1050867

[a] CTAB = cetrimonium bromide (also: hexadecyltrimethylammonium bromide).

[b] SDS = sodium dodecylsulfate.

[c] Values in brackets refer to highest resolution shell.

[d] R_{meas} = redundancy independent R-factor[16].

Table S2. Intra-helical hydrogen bonds of oligourea-surfactant co-crystal structures 1-4 plus a surfactant-free nona-urea oligourea crystal structure.

Urea carbonyl*		**Structure 1** *Oligourea **1***	**Structure 2** *Oligourea **1** (A)*	**Structure 2** *Oligourea **1** (B)*	**Structure 3** *Oligourea **2***	**Structure 4** *Oligourea **3***	**Surfactant-free oligourea** (*CCDC 750017*[8])
**1**	*H-bond 1*	2.7	2.8	2.9	2.9	2.8	3.0
	H-bond 2	2.9	2.8	2.8	2.9	2.9	3.0
**2**	*H-bond 1*	2.9	2.9	2.9	2.9	2.9	2.9
	H-bond 2	2.8	2.7	2.7	2.9	3.0	2.8
**3**	*H-bond 1*	2.9	3.0	2.9	2.9	2.8	2.9
	H-bond 2	2.9	2.9	2.9	3.0	2.9	2.9
**4**	*H-bond 1*	2.8	2.8	2.9	2.9	2.9	2.9
	H-bond 2	3.0	3.0	3.0	3.0	3.0	2.8
**5**	*H-bond 1*	2.8	2.8	2.7	2.7	2.8	2.9
	H-bond 2	2.8	2.7	2.8	2.7	2.8	2.9
**6**	*H-bond 1*	3.1	3.1	3.1	3.1	3.0	2.8
	H-bond 2	3.1	3.1	3.0	3.2	3.2	3.0
**7**	*H-bond 1*	2.9	3.1	3.1	2.9	3.0	2.8
	H-bond 2	2.8	2.9	2.9	2.8	2.8	2.8
**8**	*H-bond 1*	3.0	3.1	3.1	3.0	3.0	-
	H-bond 2	2.9	2.9	2.9	3.0	2.9	-
**9**	*H-bond 1*	3.0	3.1	3.0	3.1	3.0	-
	H-bond 2	2.9	3.0	2.9	3.0	2.9	-
**10**	*H-bond 1*	-	-	-	-	-	-
	H-bond 2	-	-	-	-	-	-
**11**	*H-bond 1*	-	-	-	-	-	-
	H-bond 2	-	-	-	-	-	-
Average intra-helical H-bond distance		2.90	2.93	2.92	2.94	2.92	2.89

*Urea carbonyls numbered consecutively from the N-terminal.

References

1 C. Douat-Casassus, K. Pulka, P. Claudon and G. Guichard, *Org. Lett.*, 2012, **14**, 3130–3133.

2 G. W. Collie, K. Pulka-Ziach, C. M. Lombardo, J. Fremaux, F. Rosu, M. Decossas, L. Mauran, O. Lambert, V. Gabelica, C. D. Mackereth and G. Guichard, *Nat. Chem.,* 2015*,* **7**, 871–878*.*

3 T. Vojkovsky, *Pept. Res.*, 1995, **8**, 236–237.

4 W. Kabsch, *Acta Crystallogr. D Biol. Crystallogr.*, 2010, **66**, 125–132.

5 M. D. Winn, C. C. Ballard, K. D. Cowtan, E. J. Dodson, P. Emsley, P. R. Evans, R. M. Keegan, E. B. Krissinel, A. G. W. Leslie, A. McCoy, S. J. McNicholas, G. N. Murshudov, N. S. Pannu, E. A. Potterton, H. R. Powell, R. J. Read, A. Vagin and K. S. Wilson, *Acta Crystallogr. D Biol. Crystallogr.*, 2011, **67**, 235–242.

6 P. Evans, *Acta Crystallogr. D Biol. Crystallogr.*, 2006, **62**, 72–82.

7 A. J. McCoy, R. W. Grosse-Kunstleve, P. D. Adams, M. D. Winn, L. C. Storoni and R. J. Read, *J. Appl. Crystallogr.*, 2007, **40**, 658–674.

8 L. Fischer, P. Claudon, N. Pendem, E. Miclet, C. Didierjean, E. Ennifar and G. Guichard, *Angew. Chem. Int. Ed. Engl.*, 2010, **49**, 1067–1070.

9 P. Emsley, B. Lohkamp, W. G. Scott and K. Cowtan, *Acta Crystallogr. D Biol. Crystallogr.*, 2010, **66**, 486–501.

10 G. N. Murshudov, P. Skubák, A. A. Lebedev, N. S. Pannu, R. A. Steiner, R. A. Nicholls, M. D. Winn, F. Long and A. A. Vagin, *Acta Crystallogr. D Biol. Crystallogr.*, 2011, **67**, 355–367.

11 A. W. Schüttelkopf and D. M. F. van Aalten, *Acta Crystallogr. D Biol. Crystallogr.*, 2004, **60**, 1355–1363.

12 P. Kumar and M. Bansal, *J. Biomol. Struct. Dyn.*, 2012, **30**, 773–783.

13 M. Bansal, S. Kumar and R. Velavan, *J. Biomol. Struct. Dyn.*, 2000, **17**, 811–819.

14 W. L. DeLano, *The PyMOL Molecular Graphics System*, DeLano Scientific, San Carlos, CA, USA, 2002.

15 R. A. Laskowski, *J. Mol. Graph.*, 1995, **13**, 323–330.

16 K. Diederichs and P. A. Karplus, *Nat. Struct. Biol.*, 1997, **4**, 269–275.
